# Facile Fabrication of Cellulose Nanofibrils/Chitosan Beads as the Potential pH-Sensitive Drug Carriers

**DOI:** 10.3390/polym14112286

**Published:** 2022-06-04

**Authors:** Meiyan Wu, Wangfang Deng, Yidong Zhang, Chao Chen, Zhexuan Liu, Pedram Fatehi, Bin Li

**Affiliations:** 1 CAS Key Laboratory of Biofuels, Qingdao Institute of Bioenergy and Bioprocess Technology, Chinese Academy of Sciences, Qingdao 266101, China; wumy@qibebt.ac.cn (M.W.); dwf0324k@163.com (W.D.); zhangyidongqust@163.com (Y.Z.); chaochen@qibebt.ac.cn (C.C.); zhexuan0122@outlook.com (Z.L.); 2 Laboratory of Natural Materials Technology, Åbo Akademi University, FI-20500 Turku, Finland; 3 Green Processes Research Centre and Biorefining Research Institute, Lakehead University, Thunder Bay, ON P7B5E1, Canada; pfatehi@lakeheadu.ca

**Keywords:** cellulose nanofibrils, chitosan, drug carrier, tetracycline hydrochloride, drug release

## Abstract

It is highly desirable to develop a safe, highly efficient, and biodegradable drug carrier with an enhanced drug transport efficiency. Cellulose nanofibrils (CNF) and chitosan (CS) composite hydrogels are promising candidate carriers with biological compatibility and non-cytotoxicity. Herein, the CNF/CS composite beads were prepared by dissolving cellulose and CS in LiBr molten salt hydrate and regenerating in ethanol. This preparation method is facile and efficient, and the obtained porous CNF/CS beads with the weight ratio of 8:2 exhibited a large specific surface area, uniform micro-nano-sized pores, strong mechanical property, and water absorption-resistance. Moreover, these beads as drug (tetracycline hydrochloride, TH) carriers showed a higher encapsulation efficiency (47.4%) at the TH concentration of 5 mg/mL in 24 h, and a higher drug loading rate (12.0%) than pure CNF and other CNF/CS beads prepared with different ratios. In addition, the TH releasing behavior of CNF/CS (8:2) beads fitted well into the zero-order, first-order, and Higuchi models under an acid condition, indicating that the drug release of these pH-sensitive beads was mainly affected by drug concentration under an acid condition. Therefore, these CNF/CS beads have great potential to be used as drug carriers for medical applications.

## 1. Introduction

Cellulose is a β-1,4 linked polysaccharide with good biodegradability and low production price and is widely used in the fields of packaging, papermaking, textiles [1,2], etc. Over the past decades, nanocellulose from natural cellulose materials, mainly including fiber-like cellulose nanofibrils (CNF) and rod-like cellulose nanocrystals (CNC), has been paid more and more attention due to their better biocompatibility, excellent mechanical properties and lower safety risk to human health [3,4]. As reported, CNF hydrogels with high porosity and good cytocompatibility could be used as controlled drug vehicles to support growth and proliferation of cells [5]. However, the drug encapsulation efficiency of pure CNF hydrogel is relatively low [6]. As is well known, the introduction of cationic polymers with amino groups, such as polyethylenimine (PEI), polydopamine (PDA), chitosan (CS), can significantly increase the absorption amount of drug through a conjugating effect [6,7,8]. However, the preparation of a CNF/PEI or CNF/PDA composite drug carrier usually requires complex chemical reactions or expensive reagents [7,9,10]. Especially, CS as an abundant natural polymer has demonstrated excellent drug loading and releasing performance, good antibacterial properties, and low toxicity, which can be used as a drug carrier to reduce inflammation and promote wound healing [11]. More importantly, CS is suitable to be a pH-responsive drug carrier for oral medicine because it can be gradually dissolved in the gastric acid of a human being, leading to the slow releasing of the drug in the stomach [12]. Furthermore, previous studies showed the increased mechanical strength and drug loading efficiency of CNF/CS composite materials by coordinating the advantages of CNF and CS [13,14]. Therefore, CNF/CS composite materials are promising candidate carriers for medical applications. 

Commonly, CNF with a large aspect ratio and specific surface area is prepared using chemical/physical treatment methods from lignocellulose, such as 2,2,6,6-tetramethylpiperidine-1-oxyl (TEMPO)-mediated oxidation, acid hydrolysis, mechanical process [15]. In our previous work, CNF was obtained through dissolving cellulose in molten salt hydrate (MSH) and regenerating in ethanol [16]. This simple fabrication process exhibited great advantages of high dissolving efficiency (105 °C, 10 min), high yield (97.5%), and cyclic utilization of molten salt. The resultant CNF with a unique structure could be employed to prepare triboelectric nanogenerators [17], film materials [18,19], absorption materials for Cr (VI) ion or formaldehyde [20,21]. 

However, there is no report on the fabrication of drug carrier with this regenerated CNF from MSH solution. In addition, the drug loading efficiency of pure CNF (TEMPO method) needs to be improved with the control of burst release and more sustained release of drugs. Therefore, in this work, the CNF/CS composite beads were fabricated using lithium bromide trihydrate (LBTH). Then, the structure and properties of the resultant beads were characterized, and the drug loading and releasing performance of the beads as drug carriers were analyzed. Also, tetracycline hydrochloride (TH) was used as the drug model due to its broad-spectrum antibiotic performance and low toxicity. This work will provide valuable fundamental knowledge for the simple preparation of the CNF-based beads for potential use in drug carrier applications.

## 2. Materials and Methods

### 2.1. Materials

Microcrystalline cellulose (MCC) and lithium bromide (LiBr, 99 wt.%) were purchased from Aladdin Reagents (Shanghai, China). Chitosan (CS, with a deacetylation degree of 85% and molecular weight of 200 kDa), and anhydrous ethanol were purchased from Sinopharm Chemical Reagent Co., Ltd., Shanghai, China. In addition, tetracycline hydrochloride (TH) and solutions of Phosphate Buffer Saline (PBS buffer) with pH of 5.0 and 7.0 were bought from Macklin Reagents (Shanghai) and Tianjin Berens Biotechnology Co., Ltd., respectively. All reagents were used without any purification. 

### 2.2. Preparation of Cellulose Nanofibrils/Chitosan (CNF/CS) Beads 

**Figure 1:**
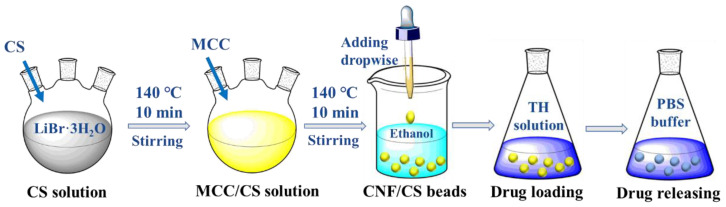
The technical route of this work.

The CNF/CS beads were prepared according to the previous report [21]. Especially, molten salt solution (LiBr·3H_2_O), was prepared by mixing lithium bromide and deionized water with the molar ratio of 1:3. Then, the molten salt solution and CS were added to a round flask, and the mixture was heated at 140 °C for 10 min in an oil bath with a stirring rate of 500 rpm. Afterward, MCC was added and heated for another 10 min to obtain a yellow mixture solution, and then MCC/CS beads were regenerated through dripping the solution into an anhydrous ethanol bath using a pipette. Here, the total amount of MCC and CS in molten salt solution was 1 wt.%, and the mass ratios of MCC to CS were 9:1, 8:2, 7:3, 6:4, and 5:5, respectively. Finally, the resultant gel beads were immersed and rinsed with running deionized water for 4 days until the conductivity of the solution containing the beads was less than 20 μs/cm. The rinsed beads were directly used as drug carriers of TH. In addition, a part of gel beads was freeze-dried using a lyophilizer (BIOCOOL, FD-1A-80, Beijing, China) at −80 °C for further characterization. The technical route of this work is shown in Figure 1.

In the absence of CS, this process would regenerate MCC with the size of <100 nm [16], thus the regenerated MCC was regarded as cellulose nanofibers (CNF), and the obtained beads were named as CNF beads (without CS). The composite CNF/CS beads (9:1, 8:2, 7:3, 6:4, or 5:5) were related to the ratios of MCC (i.e., CNF) to CS.

### 2.3. Characterization

The Fourier transform infrared spectroscopy-Attenuated Total Reflectance (FTIR-ATR) spectra of raw materials (MCC, CS and CPAM) and the resultant CNF and CNF/CS beads (8:2) were recorded using a spectrometer (Thermo Fisher, Nicolet 6700, Waltham, MA, USA) in the wavenumber range of 4000–700 cm^−1^. The crystal structure of raw materials and the resultant beads was tested by an X-ray diffractometer (Bruker, D8 ADVANCE, Germany) in the scattering angle (2θ) range of 10 and 40°. The Ni-filtered Cu Ka radiation was generated at 80 mA and 40 kV. The microstructure of the resultant beads was observed by a scanning electron microscope (SEM, Hitachi H-7600, Tokyo, Japan) with an accelerating voltage of 100 kV. All samples were coated with gold under vacuum before observation. The diameter of CNF and CNF/CS nanofibers was analyzed using a software of Nano Measurer 1.2. The data was the average of twenty tests. Brunauer-Emmett-Teller (BET) specific surface area and pore volume of CNF and CNF/CS beads were determined using a surface area and pore size analyzer (Quantachrome Instruments, Autosorb iQ, Boynton Beach, FL, USA). Total pore volume for pores was estimated from a single point on adsorption isotherm at P/Po = 0.99, and pore size was measured using the BJH method. The compressive strength and modulus of the beads were investigated using an electronic universal material testing machine (MTS Systems, CMT 6503, Shenzhen, China). Here, all CNF and CNF/CS beads for characterization were freeze-dried samples. 

### 2.4. Swelling and Degradation Properties of CNF/CS Beads

Freeze-dried CNF beads and CNF/CS beads were weighed and immersed in deionized water for 24 h, and then the free water on the surface was carefully removed with filter paper. The swelling ratio of bead samples at water saturation was calculated based on the Equation (1). In addition, the obtained CNF or CNF/CS gel beads were immersed in PBS buffer (pH = 5 or 7) for a certain time, and the mass loss ratio of the beads was calculated according to the Equation (2).

(1) $$Swelling\;ratio\;\left(g/g\right)=\frac{M_{h}-M_{d}}{M_{d}}$$

(2) $$Mass\;loss\;ratio\;\left(\%\right)=\frac{M_{h}-M_{r}}{M_{h}}\times 100\%$$

where, *M_d_* and *M_h_* are the mass of freeze-dried beads and hydrated beads, respectively, *M_r_* is the remaining mass of the hydrated beads after being immersed in PBS buffer.

### 2.5. Drug Loading Capacity of CNF/CS Beads 

Tetracycline hydrochloride (TH) was dissolved in 20 mL of deionized water under ultrasonic conditions to obtain TH solutions with different concentrations (1, 2, or 5 mg/mL). Subsequently, CNF or CNF/CS composite beads were soaked in 20 mL of TH solution for a certain time (12, 24, or 48 h, respectively). After that, the TH-loaded beads were separated through filtration, and then oven-dried at 50 °C. 

As is known, the maximum absorption wavelength of TH is 357 nm. The absorbance of TH solution with different concentrations was tested using a UV spectrophotometer (PerkinElmer Lambda 25, Waltham, MA, USA), and then the standard concentration-absorbance curve of TH solution was established. Subsequently, the concentration of TH solution after drug loading was measured by testing the absorbance of the supernatant, and the difference in the concentrations of TH before and after treating with the beads will be loaded drug. The loading ratio and encapsulation efficiency of the beads were calculated based on the Equations (3) and (4), respectively.

(3) $$Drug\;loading\;ratio\;\left(\%\right)=\frac{m_{l}}{m+m_{l}}\times 100\%$$

(4) $$Drug\;encapsulation\;efficiency\;\left(\%\right)=\frac{m_{l}}{m_{t}}\times 100\%$$

where, *m* and *m_l_* (mg) are the mass of beads and loaded drug (TH), respectively, and *m_t_* (mg) is the total mass of TH in the solution.

### 2.6. Drug Release of CNF/CS Beads In Vitro

The TH-loaded beads were placed in conical flasks and 100 mL of PBS buffer with pH of 5.0 or 7.0 were added. The flasks were incubated in a water bath with stirring speed of 50 rpm at 37 °C. After a certain time, 1 mL liquid was taken out from the flasks and the same amount of PBS buffer was supplemented in the flasks to keep the total volume of the solution unchanged. The released TH amount from beads was calculated using a standard concentration-absorbance curve, and the TH released curves of the beads were obtained. All experiments were independently repeated at least three times. The cumulative release amount of TH was calculated following the Equation (5).

(5) $$Cumulative\;release\;\left(\%\right)=\frac{100\;C_{n}+\sum^{\;}C_{n-1}}{m}\times 100\%$$

where, *m* is the mass of loaded TH of beads, *C_n_* and *C*_*n*−1_ (μg/mL) are the concentrations after TH releasing for n and n − 1 times, respectively. 

To evaluate the mechanism of TH released from the regenerated CNF or CNF/CS beads, the released data at different pH values was fitted into three commonly-used kinetic models of zero-order, first-order, and Higuchi diffusion models, respectively. The three models were described as Equations (6)–(8).

(6) $$\frac{M_{t}}{M_{\infty }}=k_{0}t$$

(7) $$\ln(1-\frac{M_{t}}{M_{\infty }})=-K_{1}t$$

(8) $$\frac{M_{t}}{M_{\infty }}=K_{2}t^{\frac{1}{2}}$$

where, $M_{t}\;$and $M_{\infty }$ are the released TH amount at time *t* and the total cumulative released TH amount, respectively, $\frac{M_{t}}{M_{\infty }}$ is the cumulation fraction of TH at time *t*, and *K*_0_, *K*_1_, and *K*_2_ are the characteristic constants of the Equations (6)–(8), respectively.

## 3. Results

### 3.1. Micro-Structure of the Resultant Beads

**Figure 2:**
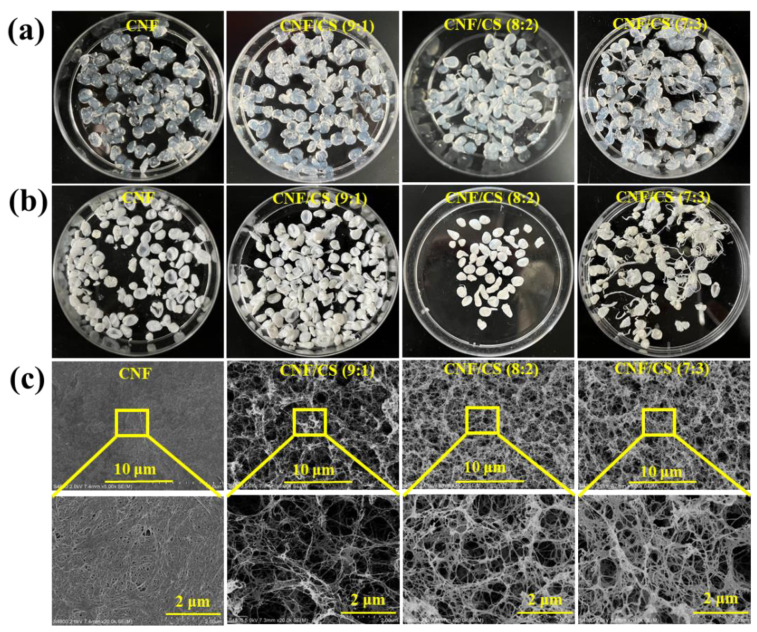
(**a**) Digital images of regenerated CNF beads and CNF/CS composite beads; (**b**) Freeze-dried CNF and CNF/CS beads; (**c**) SEM images of CNF and CNF/CS beads.

As mentioned, MCC and CS were dissolved in LiBr·3H_2_O solution, and then regenerated in anhydrous ethanol to obtain the CNF/CS composite beads. The resultant beads were used as drug carriers for TH via physical adsorption [6,7,8], and the loading capacity and release efficiency were investigated. The obtained pure CNF and CNF/CS composite beads with the mass ratio of (MCC to CS) 9:1, 8:2, and 7:3 can be seen in Figure 2a. The viscosity of MCC and CS composite solution increased with the increase in the CS amount, leading to the long tails of resultant CNF/CS beads (7:3). Especially, when the amount of chitosan was over 40%, only fiber-like composite hydrogels could be obtained in ethanol due to the hyperviscosity. Therefore, we only discussed the structure and properties of the CNF beads and the CNF/CS composite beads with the mass ratio of (MCC to CS) 9:1, 8:2, and 7:3. 

For investigating the micro-structure, these beads were freeze-dried. Due to the presence of chitosan, the color of the freeze-dried bead changed from white into light yellow (Figure 2b). Moreover, the surface of CNF bead exhibited a dense layer formed by highly aggregated CNF bundles with the average diameter of 31.8 nm (Figure 2c and Table 1). The CNF/CS beads (9:1) exhibited many large pores and individual CNF/CS nanofibers with diameter of 16.5 nm, while CNF/CS beads (8:2 and 7:3) showed more uniform pores and nanofibers with a larger diameter of 21.3 and 24.7 nm, respectively. With increasing CS amount, more CS was absorbed on the surface of regenerated CNF, leading to the increased diameter of CNF/CS nanofibers. At the same time, CS with cationic charge could enhance the electrostatic repulsion of CNF/CS nanofibers, generating the uniform dispersion of nanofibers and pores [21]. As known, the porous structure of beads, such as CNF/CS composite beads in this work, was expected to be beneficial for drug loading and slow-releasing [22]. Furthermore, the introduction of CS in CNF/CS beads could prevent shrinkage and obtain gel materials with a uniform pore size [21]. Here, the BET results showed that the specific surface area (17.9 m^2^/g) and pore volume (0.0541 cm^3^/g) of CNF/CS beads (8:2) were larger, and its corresponding pore size (12.16 nm) was smaller than other beads (Table 1), indicating the more uniform pore size distribution of these beads. Therefore, as CNF/CS beads (8:2) had larger surface area and pore volume, it is more suitable for loading drugs.

### 3.2. Characterization of CNF/CS Beads

**Figure 3:**
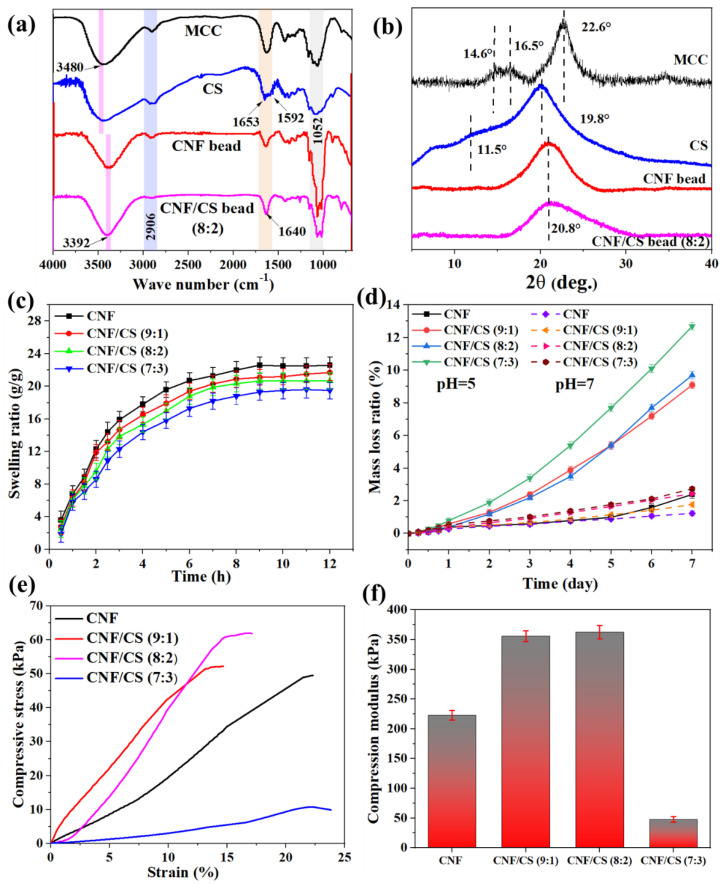
FTIR spectra (**a**) and XRD patterns (**b**) of raw materials and the obtained beads; Swelling ratio (**c**) and mass loss ratio (**d**) of CNF and CNF/CS beads; Compressive stress (**e**) and compression modulus (**f**) of CNF and CNF/CS beads.

As shown in Figure 3a, the absorption peaks at 3480 cm^−1^ correspond to the stretching vibration absorption peak of O-H bond of hydroxy groups in cellulose structure in the MCC spectrum, and this peak belongs to O-H and N-H bonds of hydroxy groups and amino groups in CS spectrum [23]. However, this vibration peak shifts to lower wavenumber (3392 cm^−1^) in the spectra of the regenerated CNF bead and CNF/CS composite beads, which is caused by the reconstruction and strengthening of hydrogen bonds between the regenerated cellulose and CS [24]. Furthermore, compared with MCC, the obvious characteristic absorption peaks of CS at 1653 cm^−1^ and 1592 cm^−1^ are attributed to amide I and amide II peaks [23], respectively. Because of the low amount of CS, only one peak can be observed around 1640 cm^−1^ in the spectrum of CNF/CS beads. In addition, the peak around 2906 cm^−1^ assigns to the stretching vibration of the saturated C-H bond, and the peak at 1052 cm^−1^ assigns to the primary hydroxyl group of the pyranoid ring in all spectra [25]. Therefore, compared with raw materials, there was no obvious change in the chemical structure of the regenerated CNF and CNF/CS beads and only the strengthening of hydrogen bond interaction influenced the properties of the regenerated beads. 

Furthermore, MCC exhibits typical cellulose I crystal structure with the main characteristic diffraction peaks of 14.6°, 16.5° and 22.6° at 2θ (Figure 3b), corresponding to the crystal structure (1–10), (110) and (200) lattice planes [16,25]. Simultaneously, the XRD pattern of CS shows characteristic crystal diffraction peaks with 11.5° and 19.8°, corresponding to (020) and (201) lattice planes [26,27]. However, the obtained CNF and CNF/CS beads after dissolving in MSH solution and regenerating in ethanol show a wide amorphous peak around 20.8°, indicating the changed crystal structure in the preparation process of CNF/CS beads. 

The swelling properties of the freeze-dried CNF/CS beads in water and the degradation properties of CNF/CS beads in PBS buffer are shown in Figure 3c,d. The swelling ratios of all beads rapidly increased at first and then remained stable, and a higher CS amount in CNF/CS beads led to a lower swelling ratio under the same swelling condition. Also, the mass loss ratio of all beads was lower than 3% after being immersed in a neutral PBS buffer for 7 h (Figure 3d). These results demonstrated that the introduction of CS could effectively improve the dimensional stability and water absorption-resistance of CNF/CS beads due to the strengthening of the hydrogen bond (synergistic actions) between CNF and CS [28]. However, the mass loss ratio of CNF/CS beads gradually increased with the increase of CS amount in acid PBS buffer (pH = 5) for the same soaking time. Especially, the mass loss (9.7%) of CNF/CS beads (8:2) was 4 times as much as that of the pure CNF beads (2.4%) for 7 h soaking. This result was because the β-1, 4-glycosidic bond of CS could be easily hydrolyzed and dissolved, thus decreasing the mass of CNF/CS beads under acid conditions [29]. 

In addition, the physical strength of the beads is a key factor to influence their application performance. As shown in Figure 3e,f, with the increase in the amount of CS, the compressive strength of CNF/CS beads firstly increased and then sharply decreased. When the weight ratio of CNF to CS was 8:2, the CNF/CS beads exhibited higher compressive strength (62 kPa), which was 1.2 and 5.8 times higher than that of the pure CNF beads and the CNF/CS beads (7:3), respectively. Simultaneously, the compression modulus of the beads also conformed with this phenomenon, and the CNF/CS beads (8:2) showed robust characteristics with a compression strength of 362 kPa. Clearly, the strengthening of hydrogen bond between CNF and CS (Figure 3a) provided significant compression resistance [30,31], while the CNF/CS beads (7:3) with excess CS exhibited an irregular shape, resulting in the non-uniform distributed stress and low compressive strength.

Therefore, owing to the excellent stability under neutral conditions and degradability under acid conditions, the robust CNF/CS beads (8:2) have great potential to be used as a drug delivery carrier as it will be degraded in the gastrointestinal tract in the presence of gastric acid for the purpose of slow-releasing of drugs.

### 3.3. Drug Loading Properties of CNF/CS Beads

**Figure 4:**
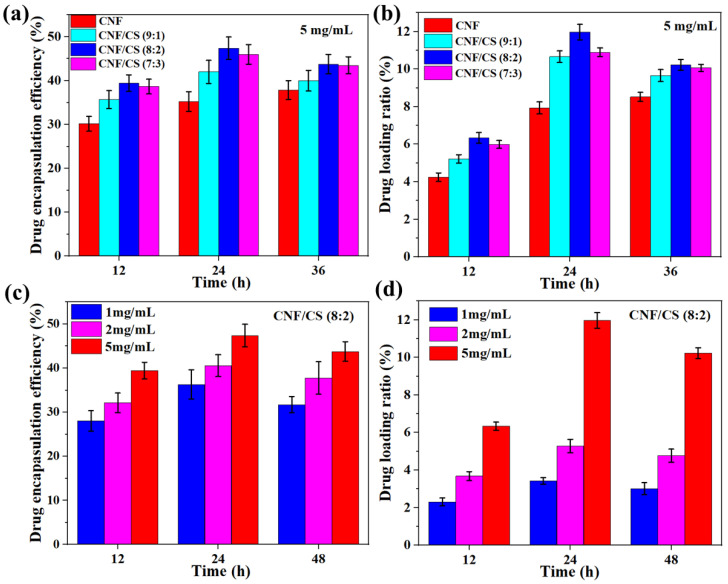
Drug encapsulation efficiency (**a**) and drug loading ratio (**b**) of CNF and CNF/CS gel beads at the TH concentration of 5 mg/mL; Drug encapsulation efficiency (**c**) and drug loading ratio (**d**) of CNF/CS beads (8:2) at different TH concentrations.

Here, TH as a broad-spectrum antibiotic with a characteristic absorption peak at 360 nm was employed to be a drug model in vitro experiment. Figure 4a shows that the drug encapsulation efficiency of the beads was 20.1–47.4% at a TH concentration of 5 mg/mL, and the encapsulation efficiency firstly increased in 24 h and then decreased from 24 to 36 h. Moreover, the encapsulation efficiency of CNF/CS composite beads was higher than that of the pure CNF beads all the time. Importantly, the encapsulation efficiency of CNF/CS beads (8:2) was the highest (47.4%) with immersing for 24 h, which was 1.9 times as much as that of the pure CNF beads. Similarly, the drug loading ratios of all beads firstly increased and then decreased with the time extension to 36 h. The drug loading ratio of the CNF/CS beads (8:2) was also the highest (12.0%) in comparison with other beads in 24 h immersion (Figure 4b). In addition, the encapsulation efficiency and loading ratio of the beads were influenced by the TH concentration (Figure 4c,d). Both encapsulation and loading ratios sharply increased with the increase in the TH concentration from 1 to 5 mg/mL at the same immersing time. For instance, the drug loading ratio of the CNF/CS beads (8:2) at the TH concentration of 5 mg/mL was 3.4 times as much as that at 1 mg/mL for 24 h. Therefore, the CNF/CS beads (8:2) with more uniform small pores (SEM images shown in Figure 2c) exhibited the higher encapsulation efficiency and drug loading ratio of TH. 

### 3.4. Drug Release Properties of CNF/CS Beads

**Figure 5:**
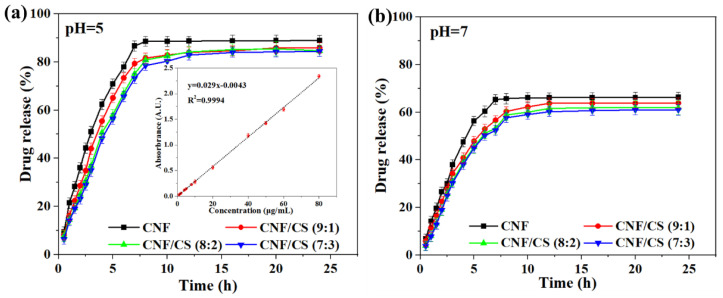
Release curves of CNF and CNF/CS beads under pH = 5 (**a**) and pH = 7 (**b**), the insert image is a standard concentration curve of TH.

According to the standard concentration curve of TH (Figure 5a, insert image), the cumulative TH release amount of the resultant beads in the buffer solution with pH values of 5 and 7 is shown in Figure 5a,b, respectively. The drug release amount of all beads at a pH of 5 increased linearly with the extension time in 7.5 h, and then remained stable from 7.5 to 24 h (Figure 5a). It indicated that the loaded TH gradually released, and there was no sudden drug release at the first stage. Moreover, compared with the TH release time (7 h) of the pure CNF beads at the maximum release amount of 88% at a pH of 5, the release time of the CNF/CS beads was relatively longer (12 h). Similarly, the release time of the CNF/CS beads at the maximum release amount was also longer compared with the pure CNF beads at pH 7, and all CNF/CS beads exhibited the similar release time and total drug release amount (Figure 5b). In addition, the TH release amount in an acidic solution (pH = 5) was much higher than that in neutral solution (pH = 7), demonstrating that the CNF/CS composite beads had a pH sensitivity. Since CNF and CS are nontoxic and safe, and chitosan can be dissolved in an acidic solution, the drug from the CNF/CS beads can be slowly released in the stomach containing gastric juice with a pH of 0.9–1.5 after taking the drug-loaded beads [12]. Therefore, the CNF/CS beads with the micro-nano pore structure formed by CNF and CS exhibited better slow-release effect than the pure CNF beads. Yet, the increase of CS had no significant obvious effect on the release time of CNF/CS beads based on the results obtained in this work. This result indicated that the overuse of CS was not needed, and the proper ratio of CNF to CS was 8:2 together with the consideration of mechanical strength of the prepared CNF/CS beads.

Also, the release amount of CNF/CS beads (8:2) in the first 1 h of releasing under acid conditions (14.5%) was lower than other reported TH carriers, such as polydopamine/CNF hydrogel (20%) [6], collagen hydrogel (42%) [32], polycarboxybetaine hydrogel (35%) [33], and hyaluronic acid/alginate scaffolds (50%) [34]. Simultaneously, the total release amount of TH from CNF/CS beads (8:2) (88%) was higher than many reported TH carriers, such as polydopamine/CNF hydrogel (77%) [6], collagen hydrogel (50%) [32], and cellulose nanocrystal (82.2%) [35]. Additionally, the release amount in the first 1 h and the total release amount of TH were comparable with the specially designed mesoporous polydopamine/graphene oxide/CNF (MPDA/GO/CNF) hydrogel with an encapsulation structure (14% and 84.3%, respectively) [36]. However, the preparation of the CNF/CS beads (8:2) was much simpler than that of the MPDA/GO/CNF hydrogel. Hence, the as-prepared CNF/CS beads (8:2) (without a burst release) were more suitable to be used as a drug carrier with good drug-releasing performance and a facile preparation process.

**Figure 6:**
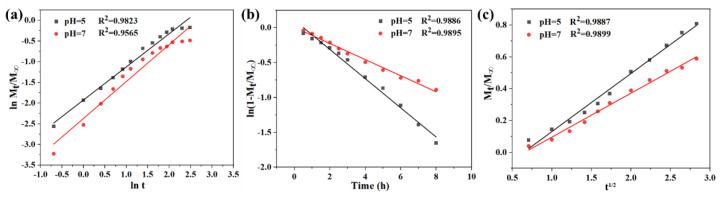
Linear fitting curves of $\ln\frac{M_{t}}{M_{\infty }}$ and lnt (**a**), $\ln(1-\frac{M_{t}}{M_{\infty }})$ and time *t* (**b**), $\frac{M_{t}}{M_{\infty }}$ and *t^1^*^/2^ (**c**) under acid and neutral conditions.

In addition, three release models of zero-order model, first-order model, and Higuchi model were used to fit the release curves of the CNF/CS composite beads (8:2) for further investigating the release mechanism (Figure 6a–c), respectively. Specifically, the zero-order kinetics model is related to drug concentration-dependent release, the first-order model describes the relationship between drug release rate and the remaining mass of the beads, and the Higuchi model is commonly used to evaluate the drug release controlled by diffusion [37]. Here, the CNF/CS beads (8:2) with the higher drug encapsulation and loading ratio were employed for model fitting, and the correlation coefficient (R^2^) indicated how good the model fitting was. Results showed that the R^2^ of all models was over 98% under acid conditions, and the R^2^ of first-order and Higuchi models (>98%) was higher than that of a zero-order model (95%) under neutral conditions. The results indicated that drug release curves of the CNF/CS beads (8:2) fitted all models well under acid condition, while fitting first-order and Higuchi models under neutral conditions. Therefore, the drug release of the CNF/CS beads with pH sensitivity was affected by drug concentration and mass of beads.

## 4. Conclusions

In this work, the CNF/CS composite beads were prepared using a facile dissolution/regeneration method, and LiBr molten salt hydrate was employed as a high-efficiency solvent of MCC and CS. When the weight ratio of MCC to CS was 8:2, the obtained CNF/CS beads exhibited a more uniform porous structure, larger specific surface area, better shape stability and stronger mechanical properties due to the synergistic action of hydrogen bonds between CNF and CS, which was beneficial for the loading and slow releasing of the drug (TH). Moreover, the drug loading and releasing investigation of the CNF/CS beads (8:2) showed higher drug encapsulation and loading efficiency (47.4% and 12.0%, respectively) in comparison with other beads. The total drug-releasing amount of the CNF/CS (8:2) was higher than 88% without burst release at pH 5. In addition, the releasing curves of the drug-loaded CNF/CS beads (8:2) fitted very well into the zero-order, first-order, and Higuchi models under an acid condition. Therefore, the as-prepared CNF/CS composite beads have the potential to be used for sustained drug delivery in medical applications.

## Figures and Tables

**Table 1:** Characterization parameters of the obtained beads.

Sample	Nanofiber Diameter (nm)	BET Surface Area (m^2^/g)	Pore Volume (cm^3^/g)	Pore Size (nm)
CNF beads	31.8 ± 6.0	3.2 ± 0.1	0.0122 ± 0.012	17.5 ± 1.1
CNF/CS beads (9:1)	16.5 ± 3.1	9.2 ± 0.1	0.0299 ± 0.009	13.3 ± 1.3
CNF/CS beads (8:2)	21.3 ± 3.4	17.9 ± 0.2	0.0541 ± 0.010	12.2 ± 1.2
CNF/CS beads (7:3)	24.7 ± 5.2	11.3 ± 0.1	0.0540 ± 0.011	16.0 ± 1.2

## Data Availability

Not applicable.
